# Genome Sequences of *Salmonella enterica* Serovar Heidelberg Isolates Isolated in the United States from a Multistate Outbreak of Human *Salmonella* Infections

**DOI:** 10.1128/genomeA.00004-12

**Published:** 2013-01-15

**Authors:** Maria Hoffmann, Yan Luo, Patricia C. Lafon, Ruth Timme, Marc W. Allard, Patrick F. McDermott, Eric W. Brown, Shaohua Zhao

**Affiliations:** a Division of Animal and Food Microbiology, Office of Research, Center for Veterinary Medicine, U.S. Food and Drug Administration, Laurel, Maryland, USA; b Division of Public Health and Biostatistics, Office of Food Defense, Communication and Emergency Response, Center for Food Safety and Nutrition, U.S. Food and Drug Administration, College Park, Maryland, USA; c Division of Foodborne, Waterborne and Environmental Diseases, Centers for Disease Control and Prevention, Atlanta, Georgia, USA; d Division of Microbiology, Office of Regulatory Science, Center for Food Safety and Nutrition, U.S. Food and Drug Administration, College Park, Maryland, USA

## Abstract

*Salmonella enterica* is recognized as one of the most common bacterial agents of foodborne illness. We report draft genomes of four *Salmonella* serovar Heidelberg isolates associated with the recent multistate outbreak of human *Salmonella* Heidelberg infections linked to kosher broiled chicken livers in the United States in 2011. Isolates 2011K-1259 and 2011K-1232 were recovered from humans, whereas 2011K-1724 and 2011K-1726 were isolated from chicken liver. Whole genome sequence analysis of these isolates provides a tool for studying the short-term evolution of these epidemic clones and can be used for characterizing potentially new virulence factors.

## GENOME ANNOUNCEMENT

Several key *Salmonella enterica* serovars are isolated repeatedly from both clinical salmonellosis cases and the retail meats/food animals associated with human infections. Among these serovars, *Salmonella* serovar Heidelberg has been determined to be the causative agent responsible for numerous human outbreaks, including invasive infections, and mortality in humans (1). Annually, this serovar causes an estimated 84,000 illnesses within the United States (1, 2). Recently, the Centers for Disease Control and Prevention investigated a multistate (six states) outbreak of *Salmonella* serovar Heidelberg infections consisting of 190 confirmed cases occurring between 1 April and 17 November 2011. Among the 154 ill persons for which information was available, 30 (19%) had been hospitalized. Collaborative investigative efforts of both state and federal public health and regulatory agencies linked this outbreak to the ingestion of “kosher broiled chicken livers” and the chopped chicken liver prepared using this product (3). Furthermore, pulsed-field gel electrophoresis (PFGE) analysis showed that the outbreak isolates had XbaI pattern JF6X01.0022. Han et al. (4) reported in 2011 that the XbaI pattern JF6X01.0022 is the most commonly noted PFGE pattern observed in *Salmonella* serovar Heidelberg.

To date, whole genome sequences of *Salmonella* serovar Heidelberg producing the JF6X01.0022 XbaI pattern are not available in GenBank. In this report, we announce the availability of four draft genomes of *Salmonella* serovar Heidelberg associated with the recent multistate outbreak linked to broiled chicken livers in the United States in 2011. The four isolates 2011K-1259, 2011K-1232, 2011K-1724, and 2011K-1726 linked to the outbreak share the JF6X01.0022 XbaI pattern as well as the JF6A26.0001 BlnI pattern. The isolates 2011K-1259 (Pennsylvania) and 2011K-1232 (New York) were recovered from human stool samples, whereas 2011K-1724 (New York) and 2011K-1726 (New York) were recovered from broiled chicken liver and chopped chicken liver, respectively.

The genomic DNA of each strain was isolated from overnight cultures using a DNeasy blood and tissue kit (Qiagen). The genomes were sequenced with a GS FLX+ 454 system (Life Sciences, Branford, CT) using a GS FLX titanium XL+ sequencing kit according to the manufacturer’s recommended protocol to generate between 15- and 30-fold coverage. *De novo* assemblies were performed using Roche Newbler version 2.6. The draft genome sequences of strains 2011K-1259, 2011K-1232, 2011K-1724, and 2011K-1726 consisted of 72, 84, 51, and 72 contigs, respectively, yielding total sequences for each isolate of 4,753,867, 4,750,315, 4,753,536, and 4,756,094 bp with N50 contig sizes of 197,381, 119,452, 231,121, and 180,754 bp, respectively. The overall GC content for the isolates was determined to be 52.08%. Sequences were annotated using the NCBI Prokaryotic Genomes Automatic Annotation Pipeline (PGAAP; http://www.ncbi.nlm.nih.gov/genomes/static/Pipeline.html) and have been deposited at DDBJ/EMBL/GenBank. A total of 4,584, 4,591, 4,565, and 4,574 coding DNA sequence (CDS) genes for 2011K-1259, 2011K-1232, 2011K-1724, and 2011K-1726, respectively, were determined.

A detailed report of a full comparative analysis of the genomes of these strains and other available *Salmonella* serovar Heidelberg strains will be included in future publications.

### Nucleotide sequence accession numbers. 

The draft genome sequences for these four *Salmonella* serovar Heidelberg strains have been deposited at DDBJ/EMBL/GenBank under the accession numbers AMBU00000000, AMBV00000000, AMBW00000000, and AMBX00000000.
